# Evaluation of cardiac functions after catheter ablation of atrioventricular nodal reentrant tachycardia

**DOI:** 10.3906/sag-2005-254

**Published:** 2021-04-30

**Authors:** Ahmet Çağrı AYKAN, Can Yücel KARABAY, Mustafa YILDIZ

**Affiliations:** 1 Department of Cardiology, Faculty of Medicine, Kahramanmaraş Sütçü İmam University, Kahramanmaraş Turkey; 2 Department of Cardiology, Siyami Ersek Education and Research Hospital, İstanbul Turkey; 3 Department of Cardiology, İstanbul Cerrahpaşa University Haseki Cardiology Institute, İstanbul Turkey

**Keywords:** Speckle tracking, AVNRT, tachycardia, strain, ablation

## Abstract

**Background/aim:**

Radiofrequency catheter ablation (RFA) is the most effective method of supraventricular tachycardia therapy. Recurrent supraventricular tachycardia causes systolic dysfunction and dilated cardiomyopathy. The aim of this study was to evaluate the long-term alterations of atrial and ventricular functions after RFAof typical atrioventricular nodal reentrant tachycardia (AVNRT).

**Materials and methods:**

This cross-sectional study included 55 consecutive patients with symptomatic drug-resistant AVNRT who had had an invasive electrophysiology study and RFA. Speckle-tracking–based echocardiographic assessment was performed shortly before and 1 year after the operation. Left ventricle (LV) and right ventricle (RV) peak systolic strain (PSS) and atrial strain measurements were performed.

**Results:**

RFA successfully eliminatedtachyarrhythmia in all patients. LV apical 4-chamber PSS –20.8% (–24.7 to –16.0) vs. –22.8% (–26.6 to –17.0, P < 0.001), LV apical 2-chamber PSS –21.5% (–26.8 to –10.1) vs. –22.0% (–27.8 to –13.7, P < 0.001), LV global PSS –20.4% (–26.4 to –14.4) vs. –23.0% (–27.1 to –2.3, P < 0.001), RV global PSS –26.0% (–30.0 to –18.0) vs. –26.5% (–32.1 to –19.7, P < 0.001), and peak left atrial longitudinal strain 41.0% (19.0–71.8) vs. 54.0% (25.6–82.0, P < 0.001) were significantly improved 1 year after RFA.

**Conclusion:**

RFA of AVNRT not only provides relief of palpitations but also improves cardiac functions.

## 1. Introduction

Radiofrequency catheter ablation (RFA) is an effective and accepted treatment strategy in supraventricular tachyarrhythmias [1–3]. Recurrent supraventricular tachycardia may cause systolic dysfunction and dilated cardiomyopathy [4]. RFA could improve ventricular functions in patients with arrhythmic cardiomyopathies [5]. Speckle tracking echocardiography techniques, including strain, strain rate (SR), and torsion measurements, provide more precise assessment of systolic and diastolic function of the heart than conventional parameters [6]. We have shown previously that RFA of atrioventricular nodal reentrant tachycardia (AVNRT) improved left atrial functions soon after the procedure [7]. However, to our knowledge, reports of the long-term alterations of cardiac functions after AVNRT RFA are lacking in the literature. We aimed to assess the long-term variation of atrial and ventricular functionsafter RFA AVNRT by usingspeckletracking echocardiography.

## 2. Methods 

### 2.1. Patients

This was a cross-sectional study including 55 successive patients who underwent an invasive electrophysiology study and RFA between April 2018 and August 2018 due to symptomatic drug-resistant typical slow-fast AVNRT.

Patients with atrioventricular block, atrial tachycardia, atrioventricular reentrant tachycardia, paroxysmal atrial fibrillation,bundle branchblock, chronic renal failure (estimated glomerular filtration rate <60 mL/min/1.73m2), hypertension, a history of coronary artery disease, left ventricular hypertrophy, heart failure with reduced ejection fraction, diabetes mellitus, or moderate to severe valvular heart disease were excluded from the study. The study protocol was approved by our university ethics committee. This study conforms to the principles of Helsinki Declaration. Written informed consent was obtained from all patients.

A Vivid E-9 cardiovascular ultrasound system (General Electric, Horten, Norway) was used for transthoracic echocardiographic examination. Echocardiography assessment was performed 24 h before and 1 year after the RFAoperation. Atrialelectromechanical coupling times, left ventricularpeak systolic strain (LVPSS), and right ventricle peak systolic strain (RVPSS) measurements were evaluated [8]. The images were acquired at a frame rate of 70–100 frame/s. Modified biplane Simpson’s method was used for the evaluation of the left ventricular ejection fraction.

**Figure 1:**
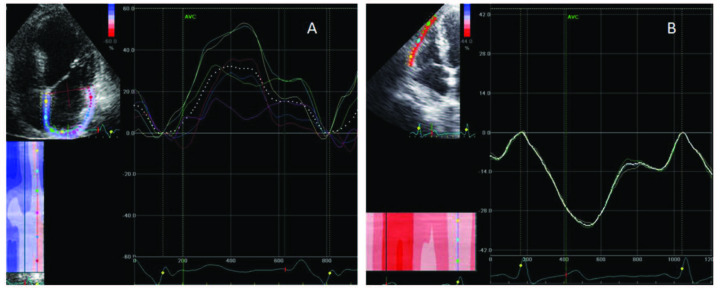
A) Measurement of left atrial strain imaging. B) Measurement of right ventricular free wall strain imaging.

The apical 4-chamber view was selected for the evaluation of left atrial deformation; peak positive longitudinal strain at the reservoir phase was determined. Right and left ventricular global longitudinal peak systolic strains were measured. EchoPAC dimension 2010 software (GE Healthcare, Chicago, IL, USA) was used by 2 experienced cardiologists to analyze echocardiographic data. The measurements of left atrial and right ventricular speckle tracking imaging are shown in Figures 1a and 1b, consecutively. 

### 2.2. Electrophysiological study and ablation procedure

Conventional electrophysiology study and RFA were performed on all patients [9]. Antiarrhythmic drugswerestopped for more than 7 days prior to RFA and discontinued thereafter. Conventional quadripolar catheter (Medtronic 6F; Medtronic, Minneapolis, MN, USA), decapolar coronary sinus catheter (Abbott Inquiry CS-5Fr; Abbott Laboratories, Abbott Park, IL, USA), His catheter (Webster His catheter; Biosense Webster, Irvine, CA, USA), and ablation catheter (7F Marinr multicurve steerable catheter; Medtronic) were introduced. A Micropace electrophysiology recorder and stimulator (Micropace SureTouch 4.0; Micropace Inc., Tustin, CA, USA) was used for programmed stimulation techniques. Typical slow-fast AVNRT was diagnosed and ablated according to standard criteria [9]. After the administration of 1 mg i.v. atropine, programmed stimulation protocols were repeated after RFA in order to confirm elimination of tachyarrhythmia. 

To standardize the frequency of AVNRT episodes,we used a scale based on history of AVNRT occurrence (1 = less than once a month; 2 = at least once a month; 3 = at least once a week). 

### 2.3. Statistical analysis

The distribution of continuous variables was evaluated with the Shapiro–Wilk test. Continuous variables were expressed as mean ± standard deviation or median (minimum-maximum), whichever was appropriate. To analyze dependent variables,the Wilcoxon signed rank test was used. Bland–Altman and intraclass observer agreement tests were used for the evaluation of inter- and intraobserver agreement. A two-tailed P < 0.05 was considered statistically significant. IBM SPSS 21.0 was used for statistical analysis (IBM Corp., Armonk, NY, USA).

## 3. Results

Programmed electrical stimulation induced AVNRT in all patients. RFA successfullyeliminated AVNRT in all patients and recurrence was not observed. Thirty-three patients were using beta blockers and 22 patients were using calcium channelblockers before the RFA. After the RFA, no patient used any antiarrhythmic drug.The mean age of the patients was 35.67 ± 6.95 years and the mean body mass index was26.48 ± 2.54 kg/m². Clinical characteristics of the patients are presented in Table 1. The duration of palpitations was 10 (2–20) years and the scale of AVNRT occurrence was2 (1–3). Apical 4-chamber LVPSS –20.8% (–24.7 to –16.0) vs. –22.8% (–26.6 to –17.0, P < 0.001), apical 2-chamber LVPSS –21.5% (–26.8 to –10.1) vs. –22.0% (–27.8 to –13.7, P < 0.001), apical long axis LVPSS –19.2% (–21.9 to –15.6) vs. –21.7% (–26.9 to –18.0, P < 0.001), global LVPSS–20.4% (–26.4 to–14.4) vs. –23.0% (–27.1 to –2.3, P < 0.001), global RVPSS–26.0% (–30.0 to –18.0) vs. –26.5% (–32.1 to –19.7, P = 0.008), peak left atrial longitudinal strain 41.0% (19.0–71.8) vs. 54.0% (25.6–82.0, P < 0.001) were significantly improved 1 year after RF catheter ablation (Table 2). Left ventricle diastolic filling velocities and left atrial volume index were also significantly improved after the procedure.

**Table 1:** The characteristics of the patients.

Age, year	35.67 ± 6.95
Male, n (%)	17 (30.9%5)
BMI, kg/m2	26.48 ± 2.54
Scale of AVNRT occurrence	2 (1–3)
Palpitation duration, year	10 (2–20)
Systolic blood pressure, mmHg	123.49 ± 8.89
Diastolic blood pressure, mmHg	75.00 ± 6.20
Procedure time, min	48 (7–80)
Fluoroscopy time, min	10 (1–21)

**Table 2:** Echocardiographic outcomes of patients before and 1 year after the procedure.

Variable	Basal	1 year follow-up	P
A4C-S, %	–20.8 [(–24.7)–(–16.0)]	–22.8 [(–26.6)–(17.0)]	<0.001
A2C-S, %	–21.5 [(–26.8)–(–10.1)]	–22.0 [(–27.8)–(13.7)]	<0.001
LAX-S, %	–19.2 [(–21.9)–(15.6)]	–21.7 [(–26.9)–(–18.0)]	<0.001
LV-G-S, %	–20.4 [(–26.4)–(14.4)]	–23.0 [(–27.1)–(–2.3)]	<0.001
RV-G-S, %	–26.0 [(–30.0)–(18.0)]	–26.5 [(–32.1)–(–19.7)]	<0.001
LA-S-r %	41.0 (19.0–71.8)	54.0 (25.6–82.0)	<0.001
TPA, ms	52.0 (25.2–71.9)	45.7 (8.0–77.0)	0.020
SPA, ms	41.0 (29.2–56.0)	47.0 (30.5–56.0)	0.078
LPA, ms	58.0 (28.3–73.0)	51.0 (23.0–69.0)	0.001
E, cm/s	65.0 (49.0–85.0)	72.0 (55.0–91.0)	<0.001
A, cm/s	72.0 (50.0–95.0)	60.0 (42.0–90.0)	<0.001
DT, ms	215.0 (164.0–263.0)	170.0 (154.0–245.0)	<0.001
LAVI, mL/m2	28.0 (20.0–35.0)	25.0 (21.0–31.0)	<0.001
LVEF, %	63.0 (55–70)	66.0 (60–70)	<0.001

A4C-S: LV apical four chamber peak systolic strain; A2C-S: LV apical two chamber peak systolic strain; LAX-S: apical long axis peak systolic strain; LV-G-S: left ventricle global peak systolic strain; RV-G-S: right ventricle global peak systolic strain; LA-S-r: peak left atrial longitudinal strain during reservoir phase; TPA: tricuspid atrial electromechanical coupling time; SPA: septal atrial electromechanical coupling time, LPA: lateral atrial electromechanical coupling time; E: peak transmitral filling velocity during early diastole; A: peak transmitral filling velocity during late diastole; DT: deceleration time of E wave; LAVI: left atrial volume index; LVEF: left ventricle ejection fraction.

**Figure 2:**
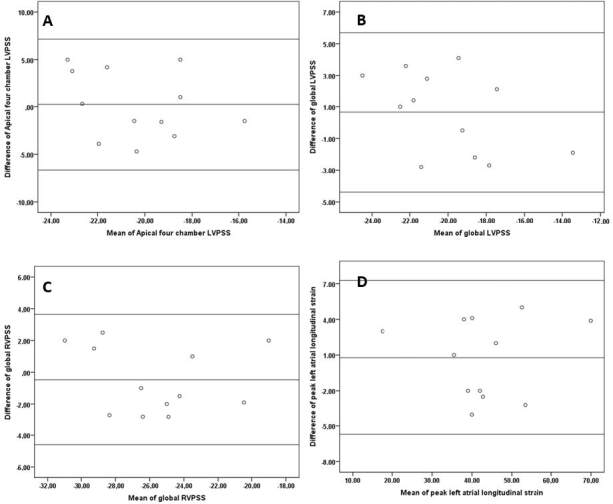
A) Bland-Altman graphic of apical four chamber peak systolic strain measurement. B) Bland-Altman graphic of left ventricle global peak systolic strain measurement. C) Bland-Altman graphic of right ventricle global peak systolic strain measurement. D) Bland-Altman graphic of left atrial peak systolic strain measurement.

Intra- and interobserver variability wereevaluated from echocardiographic data of 12 patients. To analyze interobserver variability, the second operator, who was unaware of the previous measurements, analyzed the data 2 weeks later. Two weeks after that analysis, the first operator repeated the analysis to evaluate intraobserver variability as well. The intra- and interobserver correlation of coefficient for assessment of A4C-S, A2CS, LVG-S, RV-G-S, and LA-S-r were not significantly different. A Bland–Altman graphic of apical 4-chamber peak systolic strain measurement, left ventricle global peak systolic strain measurement, right ventricle global peak systolic strain measurement, and left atrial peak systolic strain measurement are shown in Figures 2a–2d successively. 

## 4. Discussion

We found that RFA of AVNRT has long-term restorative effects on cardiac functions, as demonstrated via speckle tracking echocardiography.

RFA is the first-line therapy of AVNRT [1–3]. Recurrent AVNRT attacks may causeventricular dilatation and arrhythmic cardiomyopathy [10,11]. Myocyte loss, elongation, myofibril misalignment, and breakdown of extracellular matrix architecture were demonstrated on the cellular level [10–13].Atrial fibrillation causes atrial stunning, which ameliorates progressively after restoration of normal sinus rhythm[14]. Tachycardia attacks raiseatrial pressure, resulting in fibrosis of the atrium. Compared to atrioventricular reentrant tachycardia and atrial tachycardia, atrial pressure increase is much more prominent in AVNRT [15]. It has been shown that left atrial functions improved after RFA, along with left atrial volume reduction in atrial fibrillation/flutter patients [10–12]. Recurrent AVNRT attacks increase atrial pressure, leading to elevated atrial wall stress, which causes negative remodeling and systolic impairment [15]. Therefore, termination of AVNRT may restoreatrial and ventricular functions. Reduction of the left atriumdiameter was shown after RFA of AVNRT and AVRT [16,17]. Similarly, we found that left atrial volume was decreased after RF catheter ablation.We have recently shown that RFA of AVNRT restored left atrial functions shortly after the procedure[7]. However, long-term outcomes of AVNRT RFA on cardiac mechanics are lacking in the literature. To our knowledge, this is the first study evaluating the long-term alterations of cardiac functions after RF ablation of AVNRT. We found that peak left atrial longitudinal strain, as well as interatrial and intraatrial electromechanical coupling times, were significantly improved after RFA.

Lelakowski et al. showed that RFA improves left ventricular systolic and diastolic functions in patients with AVNRT [17]. Jimbo et al. reported that termination of AVNRT with RFA led to reduction of atrial dimensions and significantly improved exercise capacity [17]. The increase of exercise capacity wasprobably due to improved diastolic and systolic functions. Duszanskaet al. studied the variation of left ventricle systolic and diastolic functions by conventional echocardiographic parameters after AVNRT RFA. They found that successful RFA of AVNRT ameliorated left ventricle systolic and diastolic functions 6 months after the operation [18]. Similarly, we found that cardiac functions were ameliorated after RFA; however, we examined the cardiac function with speckle tracking echocardiography, a more accurate method. Additionally, our study examined the long-term effects of RFA. The evaluation of atrial functions with speckle tracking echocardiography allows assessment of entire elements of atrial functions (pump, passive conduit, and reservoir). Additionally, assessment of left ventricle functions by speckle tracking echocardiography allows detection of even subtle changes. Fishberger et al. reported that an improvement was observed in left ventricular myocardial mechanics after the RFA of ectopic atrial and permanent junctional reciprocating tachycardia. However, they did not study patients with AVNRT[19]. We found that left atrial functionsand left ventricular functionssignificantly improved after successful RFA, as assessed by speckle tracking echocardiography.

The perinodal area is an important locus of parasympathetic innervation of the heart. This area is rich in vagal fibers. Previous studies have demonstrated thatheart rate increased after RFA [20]. This increase was associated with parasympathetic withdrawal. Similarly, this partial parasympathetic withdrawal may increase both sympathetic tone and inotrophy. 

There are some limitations of this study. First, this study is relatively small in scale. MRI is a useful tool for detection of fibrosis, which is an important component of arrhythmic cardiomyopathy. Unfortunately, we did not perform an MRI study to detect fibrosis. Second, we included only patients with typical slow–fast AVNRT; however, atypical AVNRT and the left variant have the same mechanisms as typical AVNRT. Similar improvement in cardiac functions is expected to be present in atypical and left variant AVNRT cases. 

In conclusion, RFA of AVNRT not only provides relief of palpitations but also improves cardiac functions.

## Informed consent

This study conforms to the principles of the Helsinki Declaration. All patients gave informed consent. This study was approved by the Clinical Studies Ethical Committee of Kahramanmaraş Sütçü İmam University with a protocol number of 19 (28.02.2018).
